# Identification of fatty acid metabolism-related clusters and immune infiltration features in hepatocellular carcinoma

**DOI:** 10.18632/aging.204557

**Published:** 2023-03-06

**Authors:** Zhixuan Ren, Duan Gao, Yue Luo, Zhenghui Song, Guojing Wu, Na Qi, Aimin Li, Xinhui Liu

**Affiliations:** 1 Integrated Hospital of Traditional Chinese Medicine, Southern Medical University, Guangzhou 510315, China; 2 Cancer Center, Southern Medical University, Guangzhou 510315, China; 3 Department of Pharmacy, Guilin Medical University, Guilin 541004, China

**Keywords:** fatty acid metabolism, prognostic model, hepatocellular carcinoma, immune infiltration

## Abstract

Hepatocellular Carcinoma (HCC) is a type of liver cancer which is characterized by inflammation-associated tumor. The unique characteristics of tumor immune microenvironment in HCC contribute to hepatocarcinogenesis. It was also clarified that aberrant fatty acid metabolism (FAM) might accelerate tumor growth and metastasis of HCC. In this study, we aimed to identify fatty acid metabolism-related clusters and establish a novel prognostic risk model in HCC. Gene expression and corresponding clinical data were searched from the Cancer Genome Atlas (TCGA) and the International Cancer Genome Consortium (ICGC) portal. From the TCGA database, by unsupervised clustering method, we determined three FAM clusters and two gene clusters with distinct clinicopathological and immune characteristics. Based on 79 prognostic genes identified from 190 differentially expressed genes (DEGs) among three FAM clusters, five prognostic DEGs (CCDC112, TRNP1, CFL1, CYB5D2, and SLC22A1) were determined to construct risk model by least absolute shrinkage and selection operator (LASSO) and multivariate cox regression analysis. Furthermore, the ICGC dataset was used to validate the model. In conclusion, the prognostic risk model constructed in this study exhibited excellent indicator performance of overall survival, clinical feature, and immune cell infiltration, which has the potential to be an effective biomarker for HCC immunotherapy.

## INTRODUCTION

As a major health burden in the world, liver cancer is expected to affect more than one million people by 2025 [1]. The most common primary liver cancer, hepatocellular carcinoma (HCC), ranks fourth among all cancer-related deaths [1]. HCC patients in early stage can be cured by resection, transplantation, thermal ablation and TACE [2]. Early detection of HCC can increase the possibility of potentially curative treatment. Nevertheless, since early HCC diagnosis is challenging, the prognosis of HCC patients remains dismal. HCC patients with intermediate-stage can benefit from catheter-based locoregional treatment [3]. The multitargeted Tyrosine kinase inhibitors (TKI) sorafenib and lenvatinib were approved for the treatment of advanced-stage HCC [4]. A subset of patients treated with immune checkpoint inhibitors has demonstrated strong anti-tumor activity [5]. Identifying and validating predictive biomarkers is a major challenge for HCC immunotherapy. Thus, it is imperative to search novel molecular biomarkers to improve the diagnostic accuracy and guide therapies for HCC patients.

In HCC, cancer cells undergo considerable metabolic reprogramming when preparing to proliferate [6]. It has been clear that lipid metabolic rewiring is an influential metabolic alteration in cancer cells. Fatty acid is an integral component of lipid metabolism, it participates in membrane synthesis, storage of energy, and production of signaling molecules [7]. Over the past few years, there has been expanding understanding of the role of fatty acid metabolism (FAM) in tumor progression [8]. Cancer cells can obtain fatty acids from both intracellular and extracellular sources, and changes in fatty acid metabolism are characteristics of oncogenesis and metastasis [9]. By enhancing lipid synthesis, storage and degradation, aberrant fatty acid metabolism impacts the biology of cancer cells to drive tumorigenesis and disease progression [8]. A recent study has uncovered that fatty acid level influenced by cancer cell fatty acid metabolism can change CD8+ T cell activity [10]. It also found that tumor and immune cells compete for fatty acids, which promotes tumor growth [10]. According to a study, fatty acid chain lengthening has been determined as a distinguishing feature of lung cancer [11]. Increasing evidences indicated that fatty acids may contribute to the cancer initiation and development such as gastric cancer, colorectal cancer and breast cancer [12–14]. Deregulated fats can also affect the efficacy of chemotherapy and radiation therapy for cancer patients [15, 16], as well as the effectiveness of immunotherapy. Treatments that target deregulated fatty acids and the inhibition of immune checkpoints in cancer may augment each other’s effects [17]. HCC prevention and treatment may benefit greatly from an understanding of fatty acid metabolism heterogeneity, nonetheless, few studies that investigate possible mechanism and prognostic value of fatty acid metabolism-related genes (FAMs) have been conducted in HCC.

In this study, we explored the fatty acid metabolism-related clusters and assessed the composition of tumor microenvironment (TME) in HCC. First, based on expression of 49 FAMs, we identified 3 FAM clusters with distinct biological pathways and immune characteristics. Then 2 gene clusters were determined according to 190 DEGs retrieved from 3 FAM clusters. Afterward, based on the prognostic value of 190 DEGs, we established a prognostic model. Finally, the reliability of the model and the immune landscape of HCC samples were determined.

## MATERIALS AND METHODS

### Data source

On TCGA website (https://portal.gdc.cancer.gov/), gene expression information (fragments per kilobase million, FPKM) and clinical characteristics of 371 HCC patients were obtained. From ICGC database (https://icgc.org/), we acquired information of another 231 HCC patients, including RNA-seq data and clinical features [18]. Based on previous descriptions, we have transformed the LIHC (liver hepatocellular carcinoma) FPKM values into TPM (transcripts per kilobase million) values [19]. Through the GeneCards database, using “fatty acid metabolism” as a keyword, the fatty acid metabolism-related genes (FAMs) was searched and screened. Then, with a relevance score ≥ 50, 49 FAMs were retrieved for the next analyses and provided in Supplementary Table 1. In order to assess mutation states of FAM-related genes in HCC samples, mutation data was processed by “maftools” R package [20].

### Consensus clustering for FAMs

As a result of consensus unsupervised clustering analysis, HCC patients were categorized into different clusters by the R package “ConsensusClusterPlus” based on the FAMs expression [21]. Using the R packages “survival” and “survminer”, we tested whether there are any differences in survival time between distinct clusters using Kaplan-Meier curves. A heatmap plot of the clinical and pathological characteristics was created using R’s “pheatmap” package. From the MSigDB (molecular signatures database) (https://www.gsea-msigdb.org/gsea/msigdb) we extracted the hallmark gene sets (c2.cp.kegg.v7.5.1) and performed gene set variation analysis (GSVA) to determine different biological processes between distinct clusters.

### Immune landscape analysis

From previous literature, we gathered the gene sets for immune cells [22], and collected cancer-related gene signatures using the MSigDB. The level of immune cell infiltration and cancer-related gene signatures in the HCC tumor microenvironment were evaluated using single-sample gene set enrichment analysis (ssGSEA) [23]. As well, we compared expression levels of several immune checkpoints and HLA genes between different clusters.

### Analysis of DEGs

With an adjusted *p*-value of 0.001, we identified DEGs among different FAM clusters by the “limma” package in R. By using the “clusterprofiler” R package, we performed functional enrichment analyses on the DEGs to determine their potential functions and pathways. Furthermore, by using a method of unsupervised clustering based prognostic DEGs expression, HCC samples were classified into different clusters (FAM gene cluster D and FAM gene cluster E) for deeper analysis.

### Generation of the FAM-related prognostic model

Prognostic analysis of DEGs was conducted using univariate cox regression. Afterward, a prognostic model was established by lasso regression analysis and multivariate cox analysis. The risk score was calculated based on the follow formula: risk score = Σ (Expi × coefi). Expi means expression of each gene, and coefi represents the risk coefficient. HCC samples were categorized by the median risk score into high and low risk groups. Kaplan-Meier curves (K-M curves) were generated using the “survival” and “survminer” R packages in order to investigate the differences in survival between distinct groups. Based on clinical characteristics and risk scores, the “rms” package in R is used to plot the nomogram to predict survival outcomes [24]. Calibrating plot was used to determine the accuracy of the nomogram. In order to verify the model, we divided the ICGC set into high- and low-risk groups, and performed Kaplan–Meier curve and receiver operating characteristic (ROC) curve of ICGC set.

### Statistical analyses

Our statistical analyses were performed using R version 4.1.2. Differential clinical characteristics among distinct groups were analyzed by the Chi-squared test. Cox regression analysis (univariate and multivariate) was conducted to identify the independent prognostic factors. Comparison between the two groups was performed using Wilcox rank sum test. The significance level was set at *p* × 0.05, and two-tailed *p* values were applied.

## RESULTS

### Landscape of genetic variation and transcriptional alterations of FAMs in HCC

**Figure 1:**
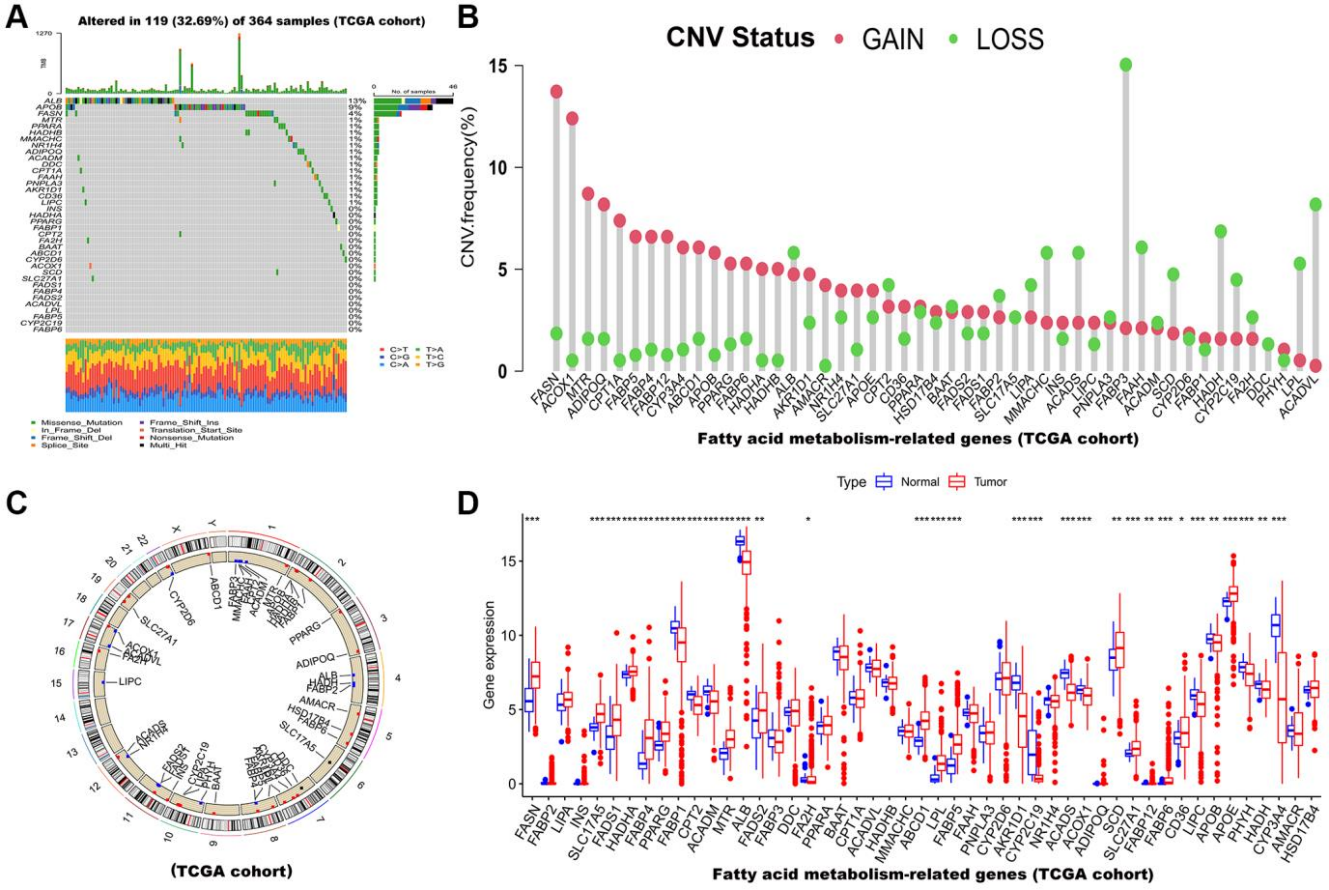
**Multi-omics landscape of FAM-related genes in HCC based on TCGA cohort.** (**A**) The mutation frequency of 49 FAMs in TCGA-LIHC cohort. Each column of the figure represents an individual patient. (**B**) The CNV variation frequency of FAMs (Red and green plots separately represent CNV gain and CNV loss). (**C**) Locations of CNV alterations in FAMs on 23 chromosomes. (**D**) The mRNA expression levels of 49 FAMs between HCC and normal tissues. Abbreviations: FAM: fatty acid metabolism; HCC: hepatocellular carcinoma; FAMs: fatty acid metabolism-related genes; TCGA: The Cancer Genome Atlas; LIHC: liver hepatocellular carcinoma; CNV: copy number variant. ^*^p < 0.05; ^**^p < 0.01; ^***^p < 0.001.

49 FAMs obtained from the Genecards website were included in this study. Based on analysis of somatic mutation incidence, the TCGA set of 49 FAMs displayed a relatively high rate of somatic mutations. FAMs mutations were detected in 119 (32.69%) of the 364 HCC samples (Figure 1A). Among these, ALB was found with the highest mutation frequency (13%), followed by APOB.

Afterward, we examined somatic copy number alterations (CNVs) in these 49 FAMs and found widespread alterations in all 49 FAMs. Among them, FASN, ACOX1 and MTR showed increased CNVs, while FABP3, ACADVL, HADH, FAAH, and ACADS showed decreases in CNVs (Figure 1B). The CNVs in the FAMs on their respective chromosomes were showed in Figure 1C. Moreover, a comparison of mRNA levels of FAMs was made between HCC tumor and normal tissues, and as showed in Figure 1D, most FAMs expression levels were positively correlated with CNV gain or loss and significantly different in tumor tissues. Consequently, while CNVs can be the primary cause of FAM expression changes, they are not the only factor that regulates mRNA expression [25]. Gene expression can also be affected by transcription factors and DNA methylation [26, 27]. We found HCC and normal samples have remarkably different genetic landscapes and mRNA expression levels of FAMs, indicating that FAMs may play an undiscovered role in HCC. Furthermore, Supplementary Figure 1A shows that the association between each FAM was highly correlated. Similarly, the infiltration levels of immune cells were assessed by ssGSEA algorithm and they showed high correlation in HCC (Supplementary Figure 1B). In summary, the above results indicated that FAMs are strongly correlated with HCC.

### Identification of FAM cluster in HCC

**Figure 2:**
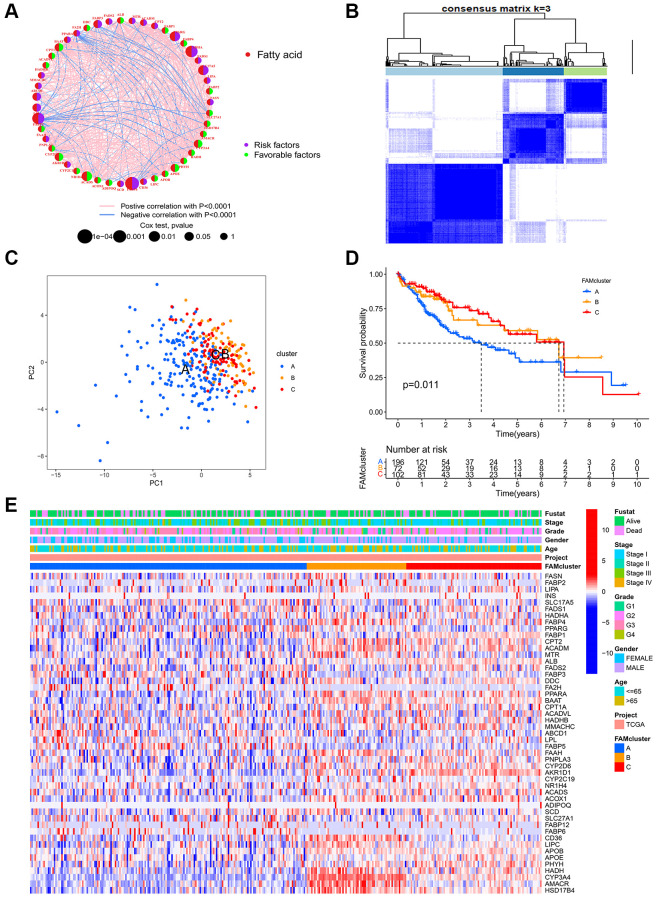
**FAM clusters and relevant clinical features.** (**A**) The interaction of expression on 49 FAMs in HCC. The line connecting the FAMs represents their interactions, with the line thickness indicating the strength of the association between FAMs. Red dots, fatty acid metabolism-related genes; Purple dots, risk factors for HCC; Green dots, favorable factors for HCC; Pink edges, positive correlation with *P* < 0.0001; Blue edges, negative correlation with *P* < 0.0001. (**B**) Consensus matrices of 49 FAMs in HCC for k = 3. (**C**) PCA analysis showing a remarkable difference in transcriptomes between the three FAM clusters in TCGA cohort. (**D**) K-M curve for the three FAM clusters. (**E**) The heatmap of clinical characteristics and expression levels of FAMs in different clusters. Abbreviations: FAM: fatty acid metabolism; FAMs: fatty acid metabolism-related genes; HCC: hepatocellular carcinoma; PCA: principal component analysis.

Through a FAMs network (Figure 2A), the full scope of FAMs interactions and their prognostic value in HCC patients was displayed. Next, consensus clustering analysis was used to investigate interactions between FAMs and HCC. Using a consensus clustering algorithm, HCC patients were categorized into different clusters (Supplementary Figure 2). Using k = 3, we were able to sort the entire cohort into cluster A (*n* = 197), B (*n* = 72) and C (*n* = 102) (Figure 2B). A principal component analysis (PCA) of the FAMs transcription profiles highlighted significant differences among the three clusters (Figure 2C). The Kaplan-Meier curves for the three FAM clusters indicated that cluster C had the most prominent survival advantage, while cluster A had the worst (*p* < 0.05) (Figure 2D). Moreover, as shown in heatmap of clinicopathological features and expression of FAMs in HCC patients, cluster A displayed the lowest level of FAMs expression (Figure 2E).

### GSVA enrichment analysis and immune infiltration estimation in distinct clusters

**Figure 3:**
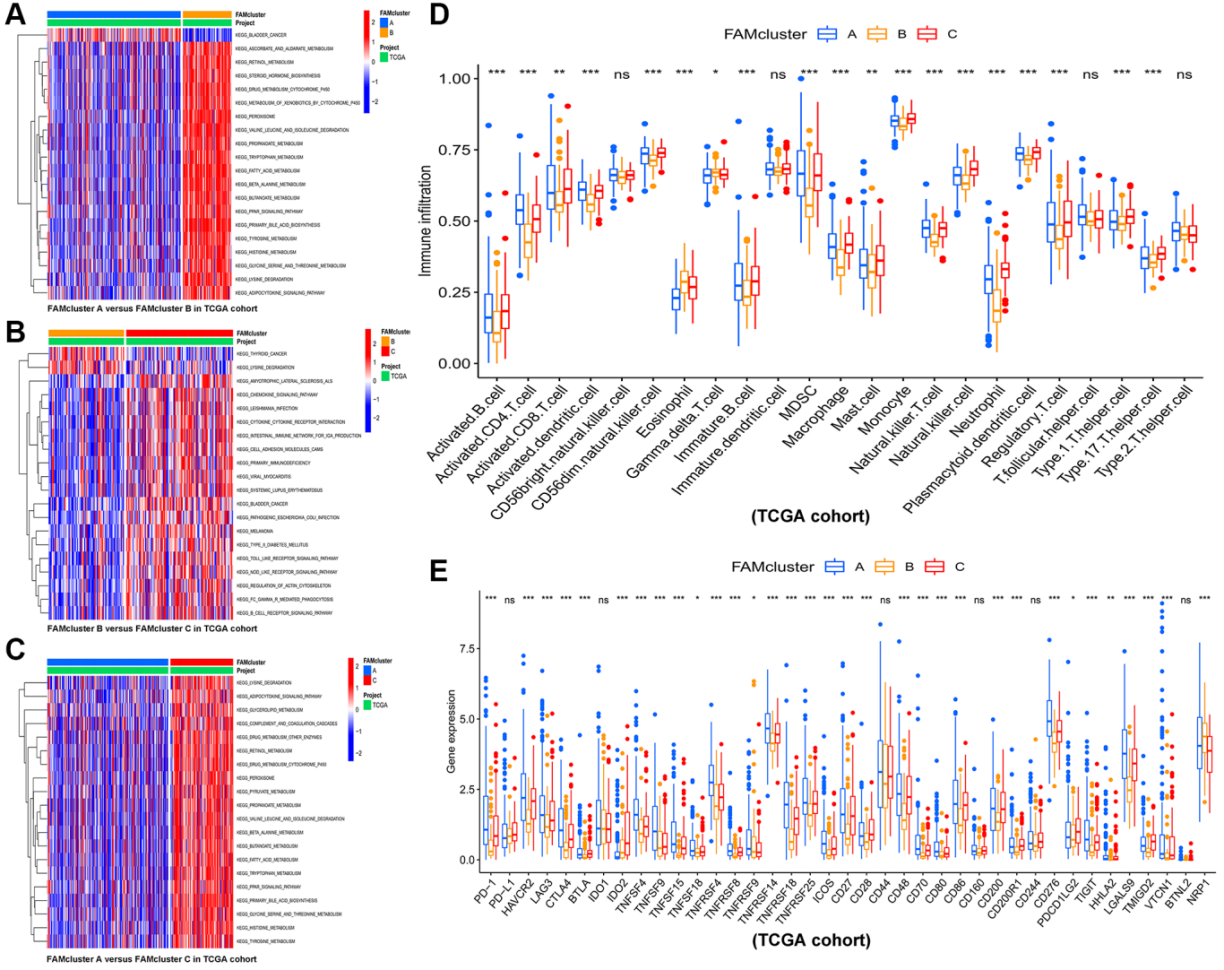
**The results of GSVA and immune infiltration analysis in three clusters.** GSVA results of biological pathways between: (**A**) cluster A vs. cluster B, (**B**) cluster B vs. cluster C, and (**C**) cluster A vs. cluster C, red and blue represent activated and inhibited pathways, respectively. (**D**) The infiltration levels of 23 immune cells in the three FAM clusters. (**E**) Significant differences in expression of immune checkpoint in the three FAM clusters. Abbreviations: GSVA: gene set variation analysis; FAM: fatty acid metabolism. ^*^p < 0.05; ^**^p < 0.01; ^***^p < 0.001.

GSVA enrichment analysis was conducted among different clusters in order to identify potential biological pathways in HCC. The top 20 pathways in each cluster were visualized (Figure 3A–3C). Cluster C was significantly enriched in fatty acid metabolism pathway and immune-related pathways, such as PPAR signaling pathway, Toll-like, B cell receptor signaling pathway, Fc-gamma-R-mediated phagocytosis pathway and Nod-like receptor signaling pathway (Figure 3C). Furthermore, in order to assess whether FAMs contribute to TME of HCC, we used the ssGSEA algorithm to calculate connection between the three clusters and 23 kinds of immune cells of every HCC sample. Among the three clusters, there were significant differences in the infiltration of immune cells (Figure 3D). Besides, HCC patients in cluster A had the highest expression level of most immune checkpoints among three FAM clusters (Figure 3E), that implied an exhausted immune TME in cluster A patients.

### Identified of gene clusters based on FAM cluster-related DEGs in HCC

**Figure 4:**
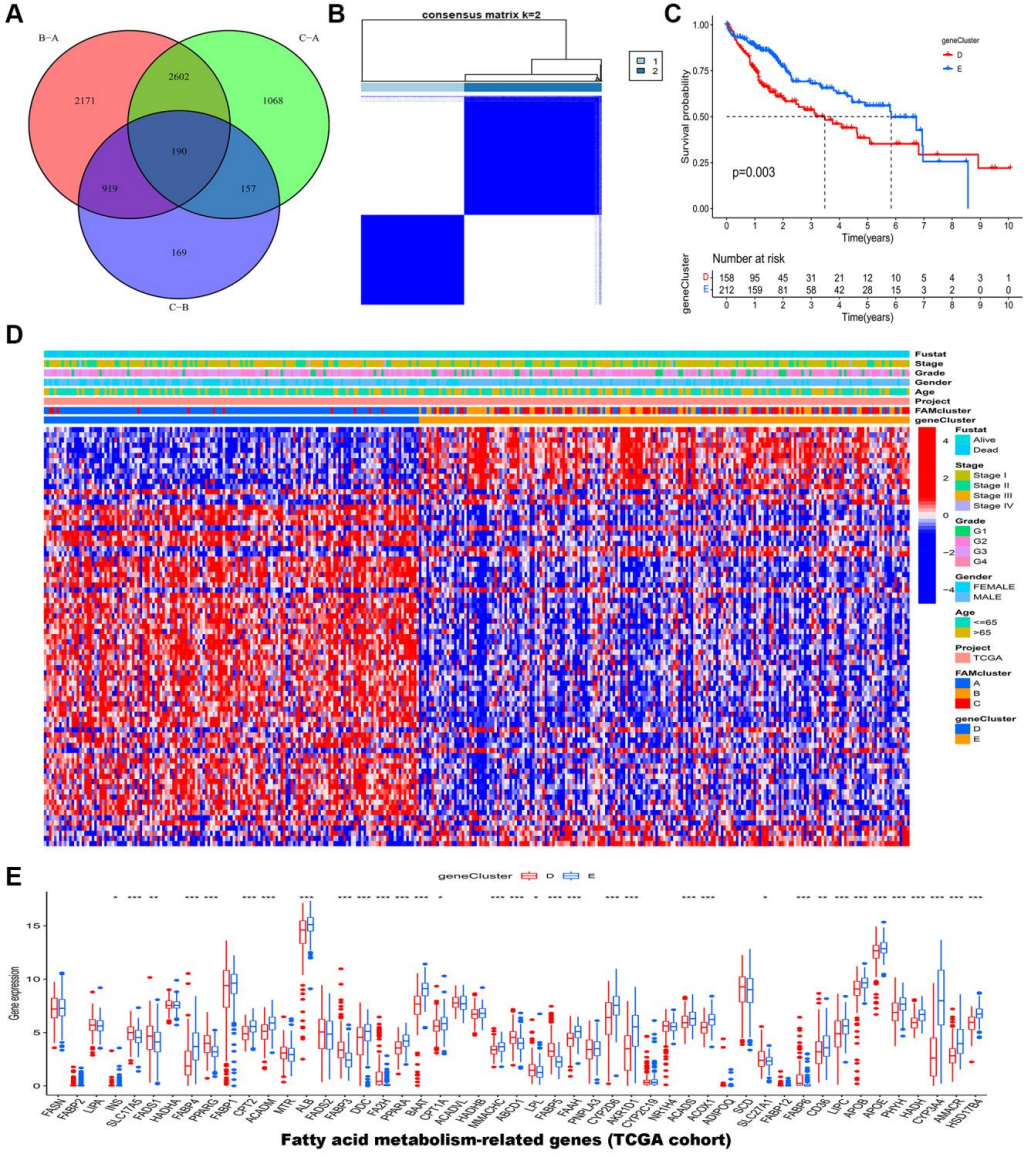
**Identification of gene clusters based on DEGs in the TCGA-LIHC cohort.** (**A**) Venn diagram showed the DEGs among the three FAM clusters. (**B**) HCC samples were divided into two clusters based on the consensus clustering (k = 2). (**C**) The OS analysis of HCC samples between gene cluster D and E. (**D**) The heatmap of clinical characteristics of HCC patients in different clusters. (**E**) The mRNA expression levels of 49 FAMs between gene cluster D and E. Abbreviations: DEGs: different expressed genes; TCGA: the Cancer Genome Atlas; LIHC: liver hepatocellular carcinoma; FAM: fatty acid metabolism; HCC: hepatocellular carcinoma; OS: overall survival; FAMs: fatty acid metabolism-related genes. ^*^p < 0.05; ^**^p < 0.01; ^***^p < 0.001.

In the previous steps, three clusters were determined, then significant DEGs with adjusted *p* value < 0.001 were identified by differential analyses between any two clusters. The Venn diagram (Figure 4A) illustrated the following intersections which resulted in 190 DEGs. A functional enrichment analysis was employed to research the potential biological behavior of 190 DEGs. According to GO (gene ontology) and KEGG (the Kyoto encyclopedia of genes and genomes) analysis, these FAM cluster-related genes were significantly enriched in metabolism pathways (Supplementary Figure 3).

Furthermore, 190 genes were screened for prognostic value by univariate cox regression analysis, and among them, 79 genes were found to be associated with overall survival (OS) in HCC (Supplementary Table 2). According to 79 prognostic genes, two genomic clusters named gene clusters D and E were identified by consensus clustering algorithm (Figure 4B and Supplementary Figure 4). According to Kaplan-Meier curves, HCC patients in gene cluster D had poorer overall survival compared to those in gene cluster E (Figure 4C). Afterward, HCC patients in FAM gene cluster D were related with higher FAM gene expression, advanced stage, advanced grade, and higher dead risk (Figure 4D). The result of further expression analysis was consistent with that in heatmap (Figure 4E).

**Figure 5:**
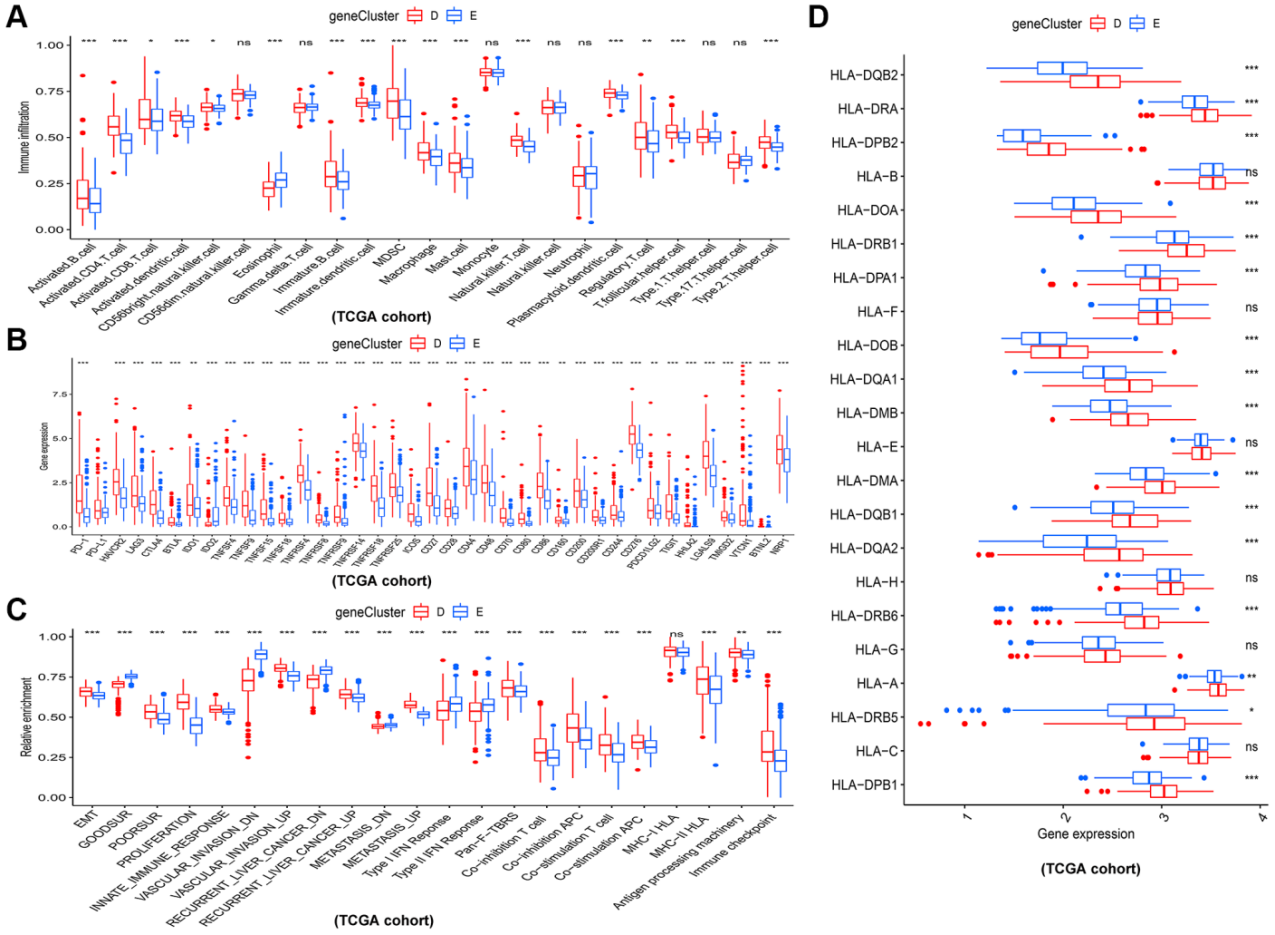
**Different immune and cancer-related characteristics in gene cluster D and E.** (**A**) The 23 kinds of immune cells in the two gene clusters. (**B**) Significant differences in expression of immune checkpoint between the two gene clusters. (**C**) The enrichment levels of cancer-related signatures in the two gene clusters. (**D**) Expression levels of HLA genes between gene cluster D and E. ^*^p < 0.05; ^**^p < 0.01; ^***^p < 0.001.

In addition, the immune analysis between two gene clusters revealed that gene cluster D tend to have higher infiltration level of most immune cells such as activated B cell, activated CD8^+^ T cells and activated CD4^+^ T cells (Figure 5A). Consistent with this, patients in gene cluster D also had higher expression level of immune checkpoints (Figure 5B). Interestedly, we estimated the relative abundance of several important cancer-related signatures by ssGSEA algorithm in different gene clusters (Figure 5C). The results showed that HCC patients in gene cluster D had higher abundance levels of bad prognostic signatures, including EMT (epithelial-mesenchymal transition), poor survival, proliferation, vascular invasion, recurrent, metastasis signatures, and immune microenvironment signatures, such as innate immune response, pan-F-TBRS, co-inhibition antigen presenting cell (APC), co-stimulation APC, co-inhibition T cell, co-stimulation T cell, MHC-I HLA (major histocompatibility complex-I human leukocyte antigen), MHC-II HLA, antigen processing machinery, and immune checkpoint, compared to those in gene cluster E. Figure 5D showed that gene cluster D had higher expression levels of HLA genes.

### Construction and verification of the prognostic risk model in HCC

**Figure 6:**
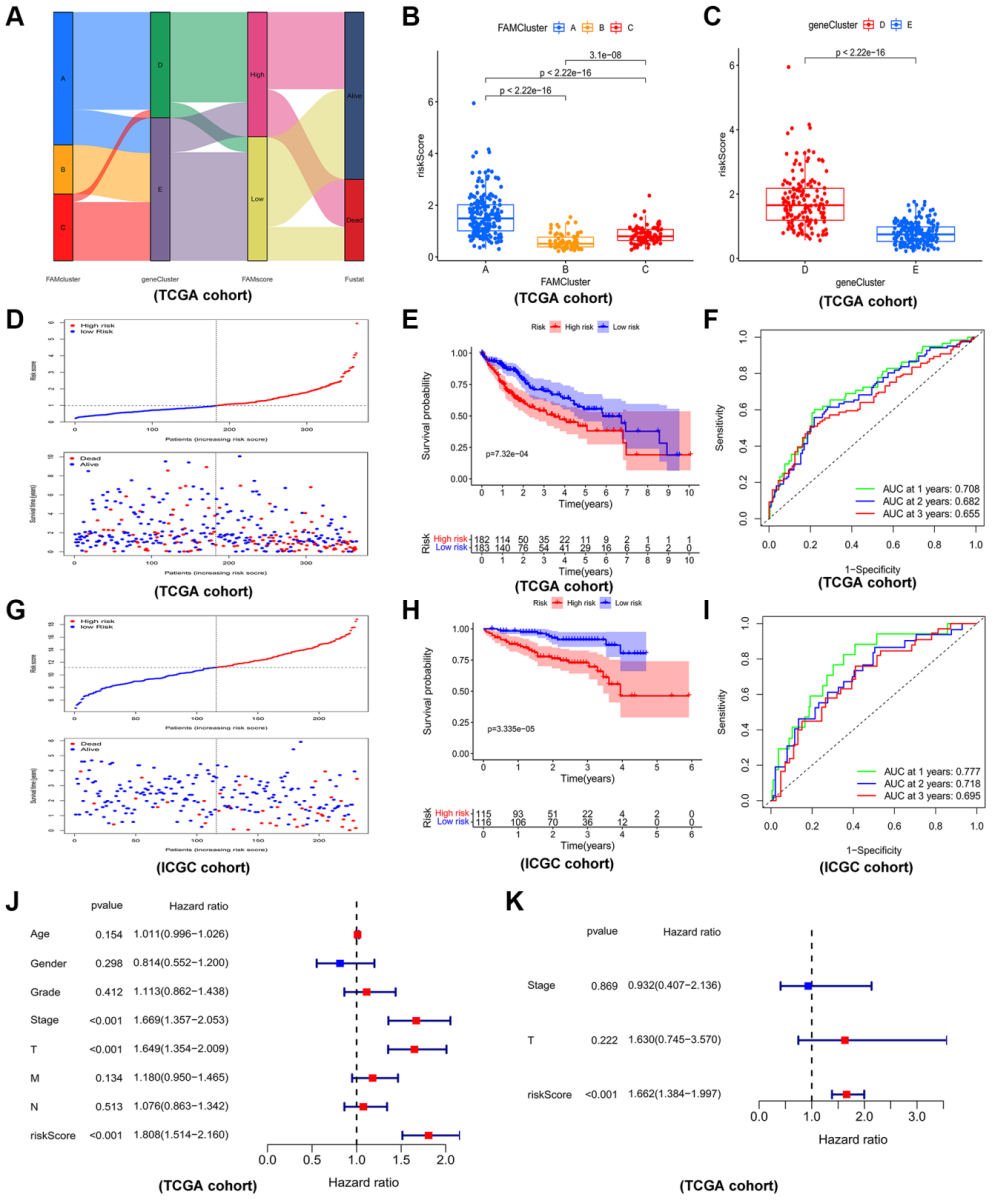
**Construction and validation of prognostic risk model.** (**A**) Alluvial diagram depicting the relationship of FAMcluster, genecluster, risk score (FAMscore) group and survival state. Boxplots of risk score in different FAMclusters (**B**) and geneclusters (**C**) Risk score distribution and scatter plots showing the risk score distribution and patient survival status in TCGA (**D**); Kaplan–Meier analysis of OS between the two groups in TCGA (**E**); ROC curves to predict the sensitivity and specificity of 1-, 3-, 5-year survival according the risk score in TCGA (**F**). Risk score distribution and scatter plots (**G**), Kaplan–Meier curves (**H**), ROC curves (**I**) of the risk model in ICGC cohort. The univariate (**J**) and multivariate (**K**) independent prognostic analysis of the model in TCGA cohort. Abbreviations: TCGA: the cancer genome atlas database; ICGC: International Cancer Genome Consortium; OS: overall survival; ROC: receiver operating characteristic.

By lasso regression analysis and multivariate cox analyses in 79 prognostic DEGs, we identified 5 genes including three risk factors (CCDC112, TRNP1, CFL1) and two protective factors (CYB5D2, SLC22A1) and created a prognostic model in HCC according to these five genes (Supplementary Figure 5 and Supplementary Table 3). The risk score of HCC patients was calculated as follows: risk score = 0.382912 × TRNP1 + 0.65021 × CCDC112 + 1.885657 × CFL1 + (−1.23099) × CYB5D2 + (−0.29032) × SLC22A1. In TCGA-LIHC set, the median cut-off value was used to stratify the patients into two groups: high-risk score (*n* = 182) and low-risk score (*n* = 183). Figure 6A displayed the distribution of HCC patients across three FAM clusters, two gene clusters, and two risk score groups. There was a significant risk score difference between FAM clusters and gene clusters. The risk score of cluster B was the lowest, while that of cluster A was the highest (Figure 6B). Cluster D had a higher risk score than cluster E (Figure 6C). In TCGA-LIHC set, high-risk patients had a worse outcome than low-risk patients, and AUC (Area under curve) values of 0.708, 0.682, and 0.650 respectively represent 1-, 2-, and 3-year survival rates of risk scores (Figure 6D–6F).

As an external validation cohort, patients in ICGC-JP (ICGC-Japan) cohort were categorized, by the median risk score, into high- and low-risk groups. Consistently, in ICGC-JP cohort, high-risk patients had worse outcomes than low-risk patients, and the corresponding AUC values of 1-, 2-, and 3-year survival rates were 0.777, 0.718, 0.695, respectively, which indicated a good efficiency (Figure 6G–6I). Furthermore, cox regression analysis, both univariate (Figure 6J) and multivariate (Figure 6K), revealed the prognostic risk model is a reliable independent prognostic factor of HCC patients.

We have done more exploration of five genes on other databases, such as TCGA (Supplementary Figure 6A–6D), ICGC (Supplementary Figure 6E–6G) and GEO database (GSE25097, GSE112790, GSE102079, GSE45267, GSE39791 datasets) (Supplementary Figure 7). Moreover, we verified IHC on HPA database (Supplementary Figure 8A) and protein expression levels on CPTAC database (Supplementary Figure 8B). Interestingly, all results are consistent with our study, which TRNP1, CCDC112, CFL1 were risk factors (compared to normal tissues, there was a significant upregulation of TRNP1, CCDC112, CFL1 expression in HCC tissues. K-M curves showed that upregulated TRNP1, CCDC112, CFL1 were associated with poor OS) and CYB5D2, SLC22A1 were protective genes (expression of CYB5D2, SLC22A1 were decreased in tumor tissues, and higher expression of CYB5D2, SLC22A1 was associated with good OS).

### Relationship of TME and the prognostic risk model in HCC

**Figure 7:**
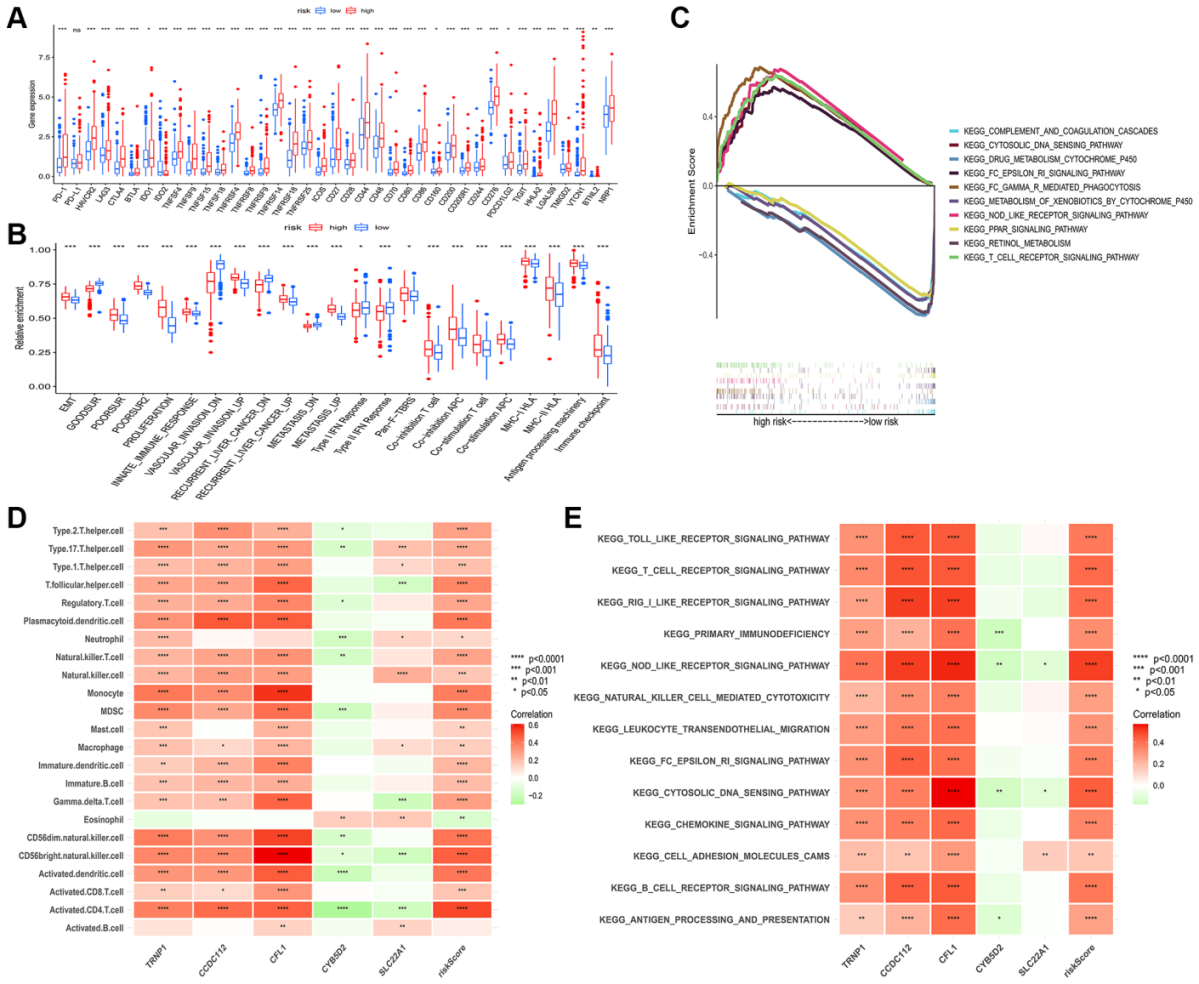
**Connection among prognostic risk model and immune or cancer-related characteristics of HCC patients.** (**A**) Significant differences in expression of immune checkpoints between the two groups. (**B**) The enrichment level of cancer-related signatures in the two groups. (**C**) Immune-related pathways enriched in the high-risk group. The correlation between genes in prognostic risk model and the infiltration level of 23 immune cells (**D**) and immune-related pathway (**E**). Red for positive associations and green for negative associations. Abbreviation: HCC: hepatocellular carcinoma. ^*^p < 0.05; ^**^p < 0.01; ^***^p < 0.001.

In the TCGA-LIHC cohort, we assessed the abundance of immune cells and cancer-related signatures by using the ssGSEA algorithm. Through the spearman method, the association among risk score and immune cells, cancer-related signatures levels were evaluated. As shown in the boxplots, the levels of immune cells (Supplementary Figure 9) and immune checkpoints (Figure 7A) in high-risk patients were higher than low-risk patients. Moreover, Figure 7B showed that high-risk patients also had higher abundance levels of bad prognostic signatures, such as EMT, poor survival, proliferation, vascular invasion, recurrent, metastasis signatures, and immune microenvironment signatures, such as innate immune response, pan-F-TBRS, co-inhibition APC, co-inhibition T cell, co-stimulation APC, co-stimulation T cell, MHC-I HLA, MHC-II HL, antigen processing machinery, and immune checkpoint compared to low-risk patients. Also, we conducted gene set enrichment analysis (GSEA) of HCC patients in different risk groups, and the result showed that high-risk group was enriched in Fc gamma R mediated phagocytosis, T cell receptor signaling pathway, Nod-like receptor signaling pathway, Fc epsilon Ri signaling pathway, while low risk group was enriched in PPAR signaling pathway and drug metabolism pathway (Figure 7C).

Furthermore, the relationship between five genes in the model and immune cells was analyzed (Figure 7D). We observed that three high-risk genes (CCDC112, TRNP1 and CFL1) were significantly positively correlated with most immune cells, whereas significant negative correlation was observed between two low-risk genes (CYB5D2 and SLC22A1) and infiltration of immune cells. Consistently, Figure 7E displayed the result of correlation between five genes, risk score and immune related pathways.

### Construction of nomogram in HCC

**Figure 8:**
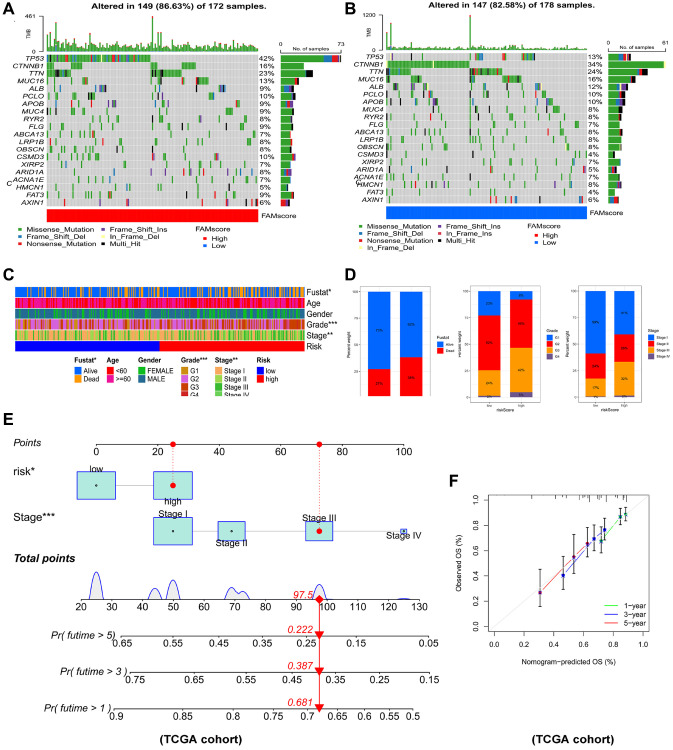
**Connections between prognostic risk model and clinical characteristics of HCC patients.** The waterfall plot of tumor somatic mutation established in (**A**) high risk group and (**B**) low risk group. (**C**) The heatmap of the model and clinical characteristics in TCGA-LIHC cohort. (**D**) Stacked bar plot of HCC survival state, pathological grade and tumor stage. (**E**) Nomogram for predicting the 1-,3- and 5- year OS of HCC patients. (**F**) Calibration curve of the program for predicting of 1-,3- and 5-year OS of HCC patients.

Comparison of genes mutations between the two risk groups revealed that high-risk patients had significantly higher mutation rates of TP53, MUC4, FLG, CSMD3, ARID1A, FAT3 than low-risk patients (Figure 8A, 8B). Moreover, high-risk HCC patients were remarkably associated with worse outcome, more advanced tumor stage and worse pathological grade (Figure 8C, 8D). To identify the reliability of this risk model in HCC patients, the prognostic nomogram plot containing the risk score and stage was constructed in TCGA-LIHC cohort (Figure 8E). Furthermore, calibration plot indicated excellent agreement between prediction and actual risk (Figure 8F). Overall, the risk model showed good prognostic value in HCC samples.

## DISCUSSION

HCC seriously threatens human health with high mortality rate. While HCC can be managed with multiple treatments, patients with the disease have extremely low 5-year survival rates due to the fact that it is commonly diagnosed in advanced stages [28].

Currently, a number of immune checkpoint inhibitors (ICIs) have been approved by the FDA (Food and Drug Administration) to treat advanced HCC, including nivolumab [29] and pembrolizumab [30]. However, there are numerous disadvantages of ICI treatment, including low response rates and side effects. Therefore, new therapeutic targets and novel prognostic models are essential for HCC patients.

Metabolism reprogramming is critical for tumor initiation and progression, especially during HCC development [31]. Synthesis of fatty acids has been involved in energy metabolism and membrane production of tumor cells. Deregulated fatty acid metabolism has been regarded as a vital metabolic regulator in supporting cancer cell proliferation [32]. A remolded microenvironment caused by abnormally fatty acid metabolism could promote HCC progression. In this study, our objective was to assess the association of FAMs and the risk of HCC.

First, we explored the mutation and correlation state of 49 FAMs obtained from the Genecard database. The top three frequently mutated genes were ALB, APOB, and FASN. Missense mutation and C>T of FAMs were the most common mutations in HCC. Due to the high expression of ALB (20%) [33] and APOB’s ability to facilitate VLDL secretion [34] (which consumes large amounts of energy), mutation of ALB or APOB may be inactivated to divert energy into cancer-relevant metabolic pathways [35]. According to the expression profiles of 49 FAMs, we determined 3 FAM clusters. Among 3 FAM clusters, cluster C had highest level of immune infiltration. Subsequently, differential analyses among 3 FAM clusters were employed. We screened 190 DEGs and showed them in a Venn plot. Based on the expression of 79 prognostic genes identified from 190 DEGs, HCC patients were grouped into 2 different gene clusters. Gene cluster D had worse survival rate, higher expression level of FAMs, and higher infiltration level of immune cells. Interestingly, gene cluster D also had higher enrichment of poor prognostic signatures, such as poor survival, liver cancer recurrent related signatures, cancer progression related signatures such as EMT, proliferation, vascular invasion, metastasis signatures, and several immune signatures, such as innate immune response, pan-F-TBRS, co-inhibition APC, co-inhibition T cell, co-stimulation APC, co-stimulation T cell, MHC-I HLA, MHC-II HLA, antigen processing machinery and immune checkpoint related signatures. These results indicated that FAMs appear to affect TME of HCC.

Moreover, based on 79 prognostic FAMs, a FAM-related model containing 5 genes (TRNP1, CCDC112, CFL1, CYB5D2, SLC22A1) was constructed by LASSO and multivariate Cox regression analysis in TCGA-LICH cohort. And we successfully confirmed the model using ICGC-JP cohort. HCC patients were categorized into two groups, high risk and low risk group. In both the TCGA and ICGC cohorts, the K-M curves showed that patients in the low group had better outcomes than those in the high group. The 1 year AUC of the model was 0.708, 0.777 in TCGA and ICGC cohort, respectively, which demonstrated that the accuracy of the risk model was excellent. The relation of our model and immune infiltration was also assessed. And the infiltration levels of immune cells were evaluated by ssGSEA. The analysis of relationship revealed that risk score was significantly positively correlated with infiltration of immune cell in HCC patients, especially CD56 bright natural killer cell, activated CD4 T cell and activated dendritic cell. The results of correlation analysis between each gene in model and HCC immunity were consistent with the properties of genes. For example, TRNP1, CCDC112, CFL1 are risk factors, then they were positively correlated with the infiltration levels of most of immune cells, whereas the results of CYB5D2, SLC22A1 were contrary to this. Patients in high risk group had higher enrichment level of poor prognostic signatures, such as poor survival, liver cancer recurrent related signatures, cancer progression related signatures such as EMT, proliferation, vascular invasion, metastasis signatures, and several immune signatures, such as innate immune response, pan-F-TBRS, co-inhibition APC, co-inhibition T cell, co-stimulation APC, co-stimulation T cell, MHC-I HLA, MHC-II HLA, antigen processing machinery and immune checkpoint related signatures.

TRNA1, CCDC112, and CFL1 expression were substantially higher in HCC tissues than in normal tissues, whereas CYB5D2 and SLC22A1 expression were significantly lower. These results were found in the TCGA, ICGC, GEO, HPA, and CPTAC databases. Liu et al. reported TRNP1 as a risk factor of four-gene model for predicting OS in HCC patients [36]. TRNP1 is essential for neural development and cell self-renewal [37]. As a hypoxia-responsive gene, CFL1 contributes to hypoxia-induced HCC progression by activating PLD1/AKT signals [38]. In a mechanism study, knockdown of CFL1 increased F-actin levels and disrupted the balance between F-actin and G-actin, which resulted in aggressiveness inhibition of HCC cells [39]. Researches have reported that decreased level of CYB5D2 is associated with breast cancer progression [40]. SLC22A1 downregulation correlates with worse patient outcomes and tumor progression [41]. It is thought that the development of HCC is accompanied by aberrant SLC22A1 variants, which may greatly affect the sorafenib levels in the affected intracellular concentrations in HCC [42]. However, there is still a lack of knowledge about how TRNP1 and CCDC112 affect the development and prognosis of HCC.

In recognition of the clinical utility of the model in predicting over survival in HCC patients, using the risk score and stage together, a nomogram was created to predict the 1-, 3-, and 5-year survival rates for HCC in TCGA cohort. The calibration plot verified the accuracy of nomograms.

Nevertheless, our study has several shortcomings. First, molecular mechanisms of these genes need to be uncovered by additional functional experiments. Second, an additional experiment is needed for further verification of model genes. Finally, since the study was analyzed on data from public database, the risk model needs to be validated by our own clinical cohort.

Taken together, we identified 3 FAM clusters, 2 gene clusters and established a novel 5-gene prognostic model for HCC patients. Fatty acid metabolism-related genes exhibited synergy with immune activation. We hope the prognostic model may help improve immunotherapy for HCC in the future.

## CONCLUSION

Our findings investigated molecular cluster and prognostic model about fatty acid metabolism in HCC, and highlighted a potential strategy for targeting the immunometabolism of HCC.

## Supplementary Materials

Supplementary Figures

Supplementary Tables
