# Supplementary material for: Identification of fatty acid metabolism-related clusters and immune infiltration features in hepatocellular carcinoma
Source: Aging (Albany NY). 2023 Mar 6;15(5):1496–523. doi: 10.18632/aging.204557 (PMC10042688; doi:10.18632/aging.204557)
Supplement: Supplementary Figures [file aging-15-204557-s001.pdf]

SUPPLEMENTARY FIGURES

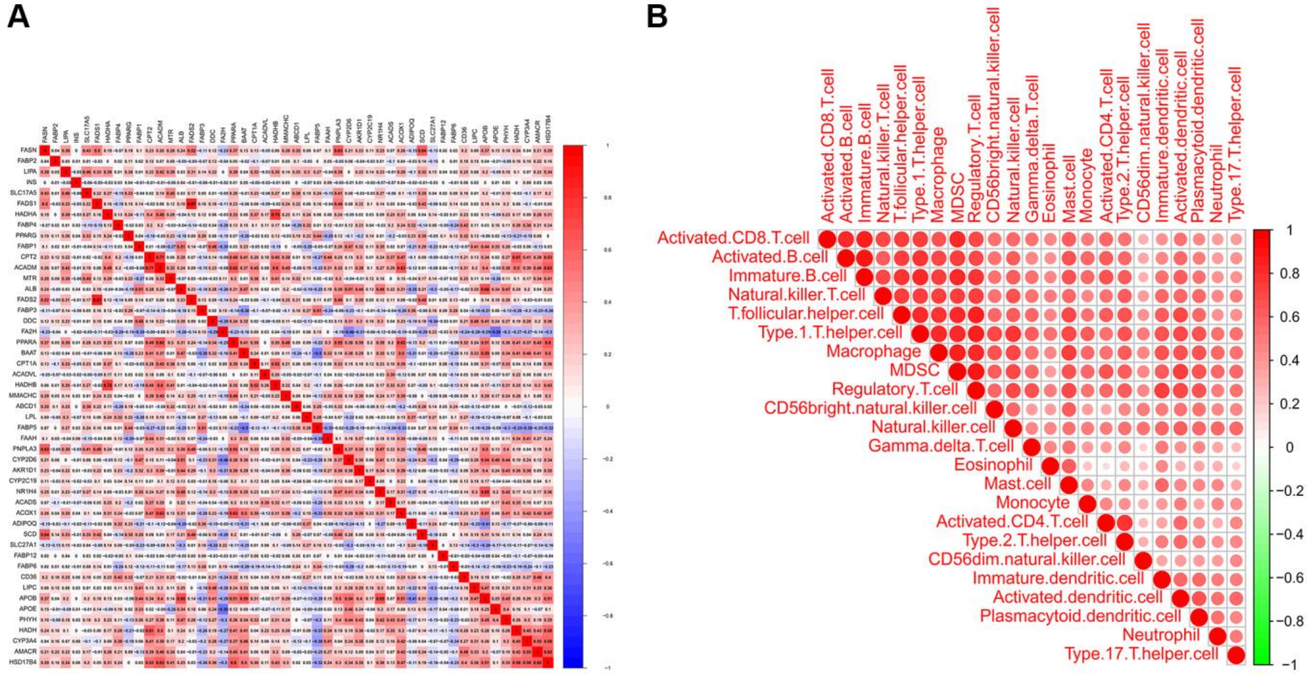

Supplementary Figure 1. The results of correlation analysis in HCC. (**A**) Correlation analysis among 49 FAMs in HCC patients. Red represents positive correlation; blue represents negative correlation. (**B**) The correlations of immune cells in HCC patients. Red dots denoting activated pathways and blue dots denoting inhibited pathways.

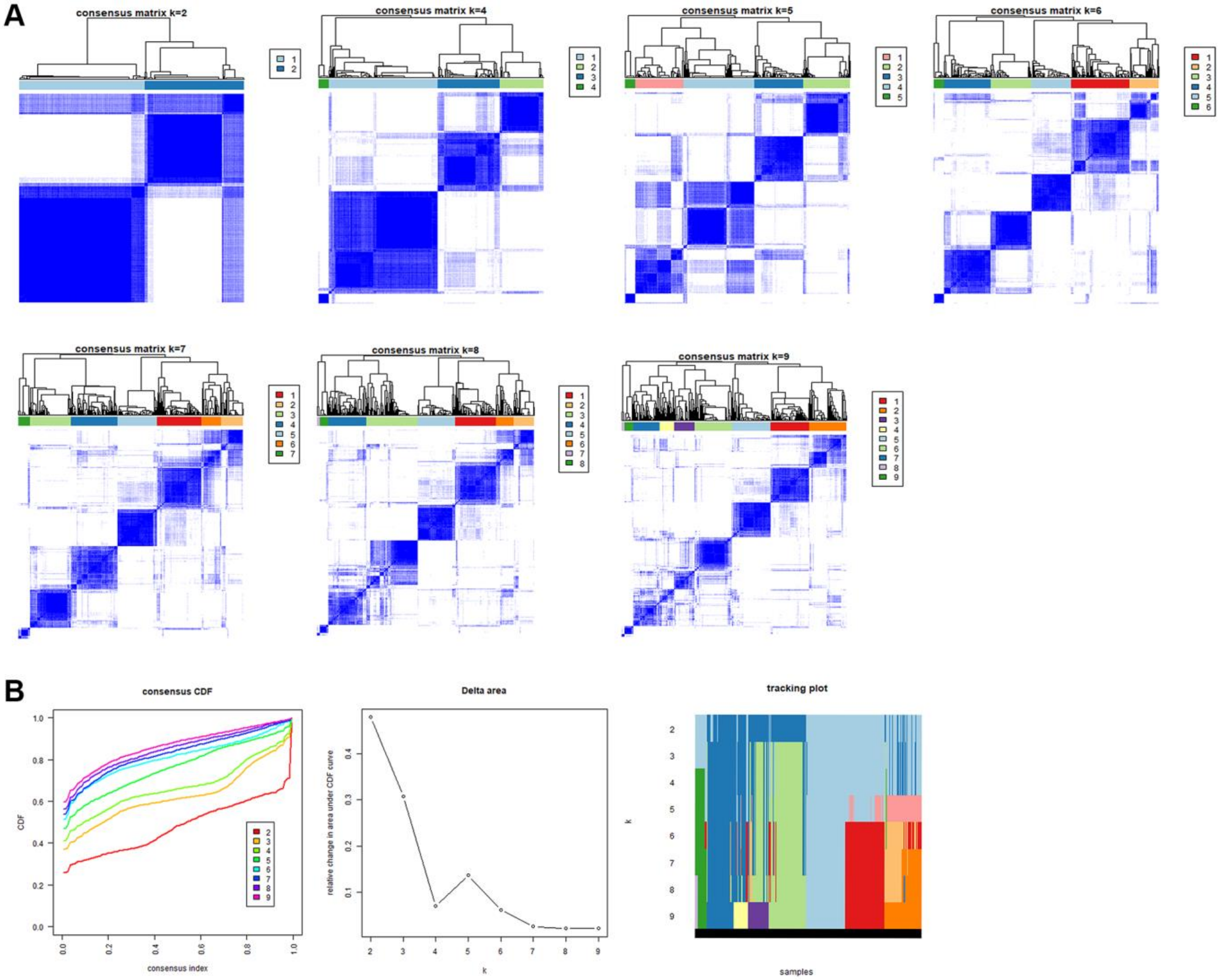

Supplementary Figure 2. The supplementary results of unsupervised clustering analysis based on 49 FAMs. (**A**) Consensus matrices of 49 FAMs in HCC. (**B**) The cumulative distribution function (CDF), relative change in area under the CDF curve, and tracking plot.

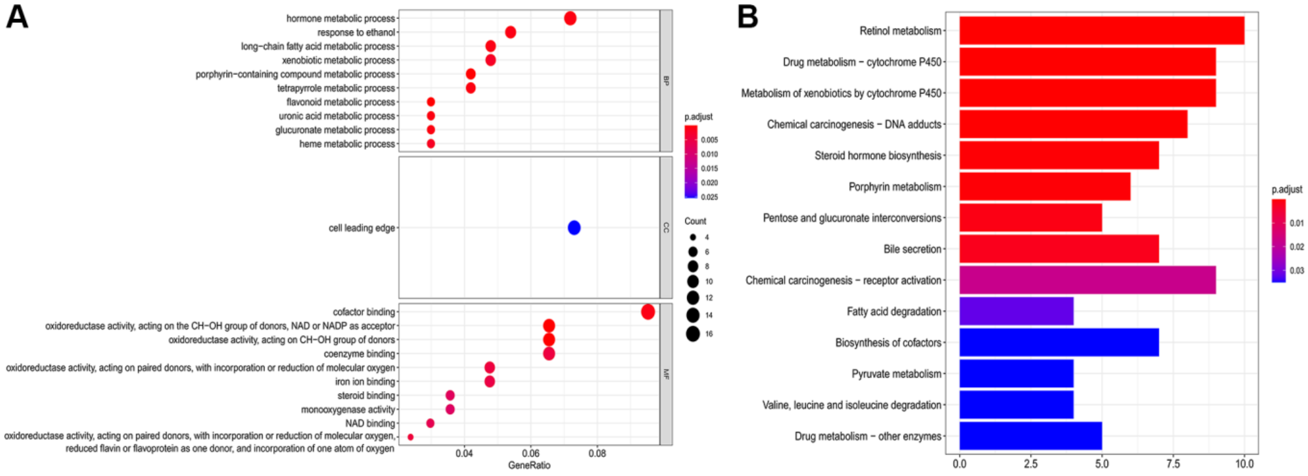

Supplementary Figure 3. GO (**A**) and KEGG (**B**) analysis of 190 DEGs among three FAM clusters.

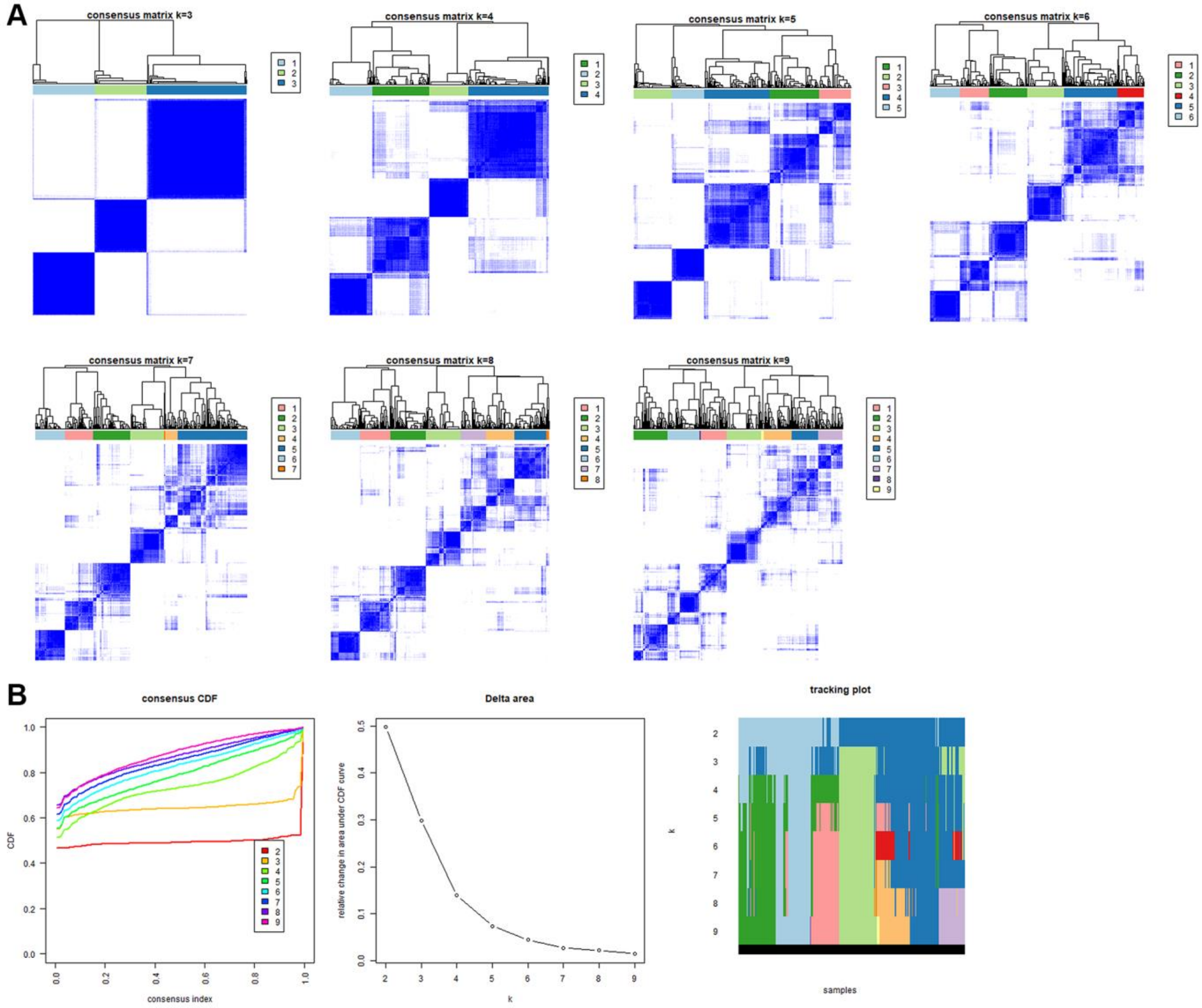

Supplementary Figure 4. The supplementary results of unsupervised clustering analysis based on 79 prognostic DEGs. (**A**) Consensus matrices of 79 prognostic DEGs in HCC for k = 3~9. (**B**) The cumulative distribution function (CDF), relative change in area under the CDF curve, and tracking plot.

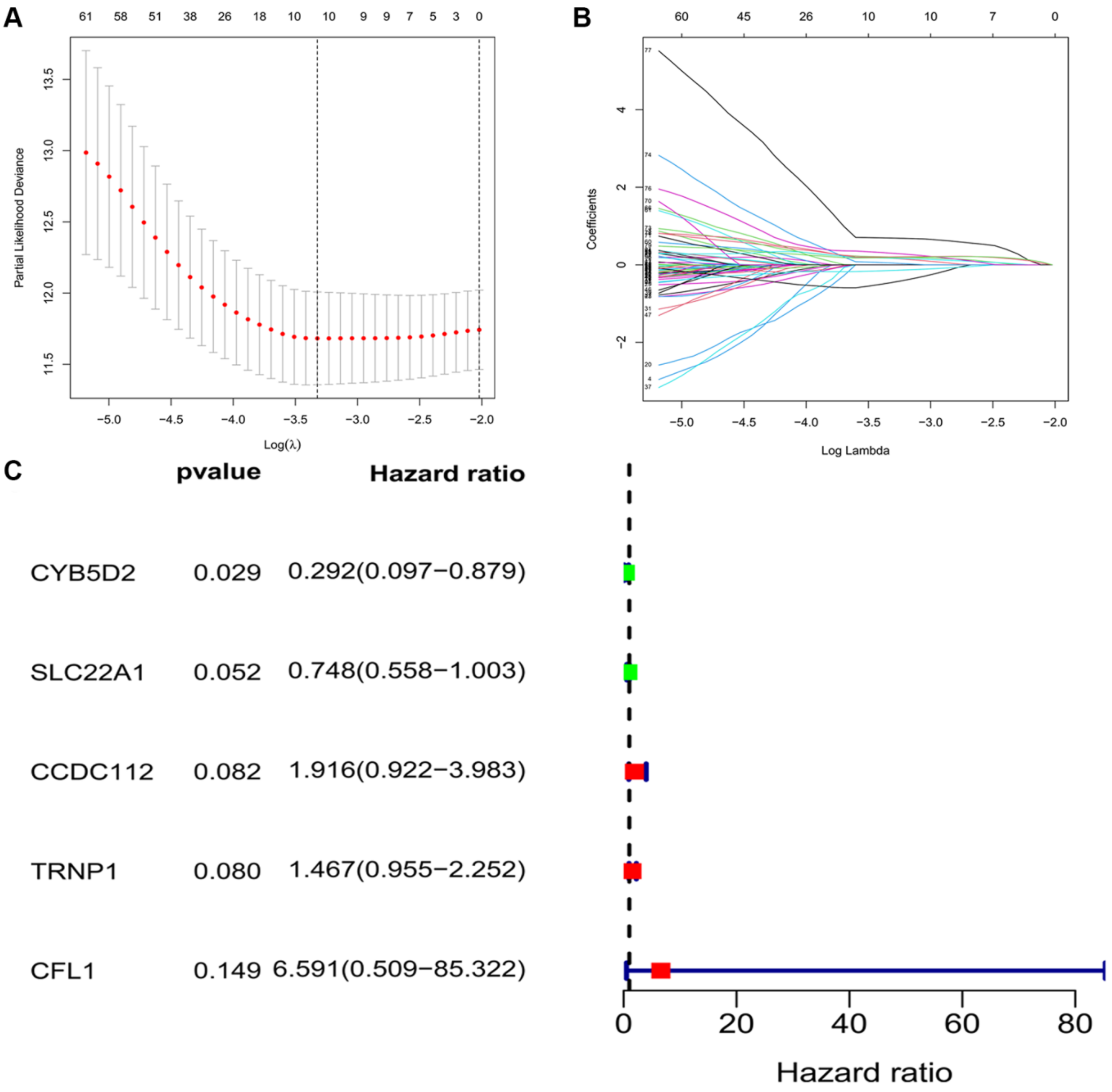

Supplementary Figure 5. Construction of risk prognostic model. (**A**) Screening of optimal parameter (lambda) at which the vertical was drawn. (**B**) LASSO coefficient profiles of the 10 DEGs with non-zero coefficients determined by the optimal lambda. (**C**) Multivariate Cox regression of five candidate genes. Green represents low-risk genes and red represents high-risk genes.

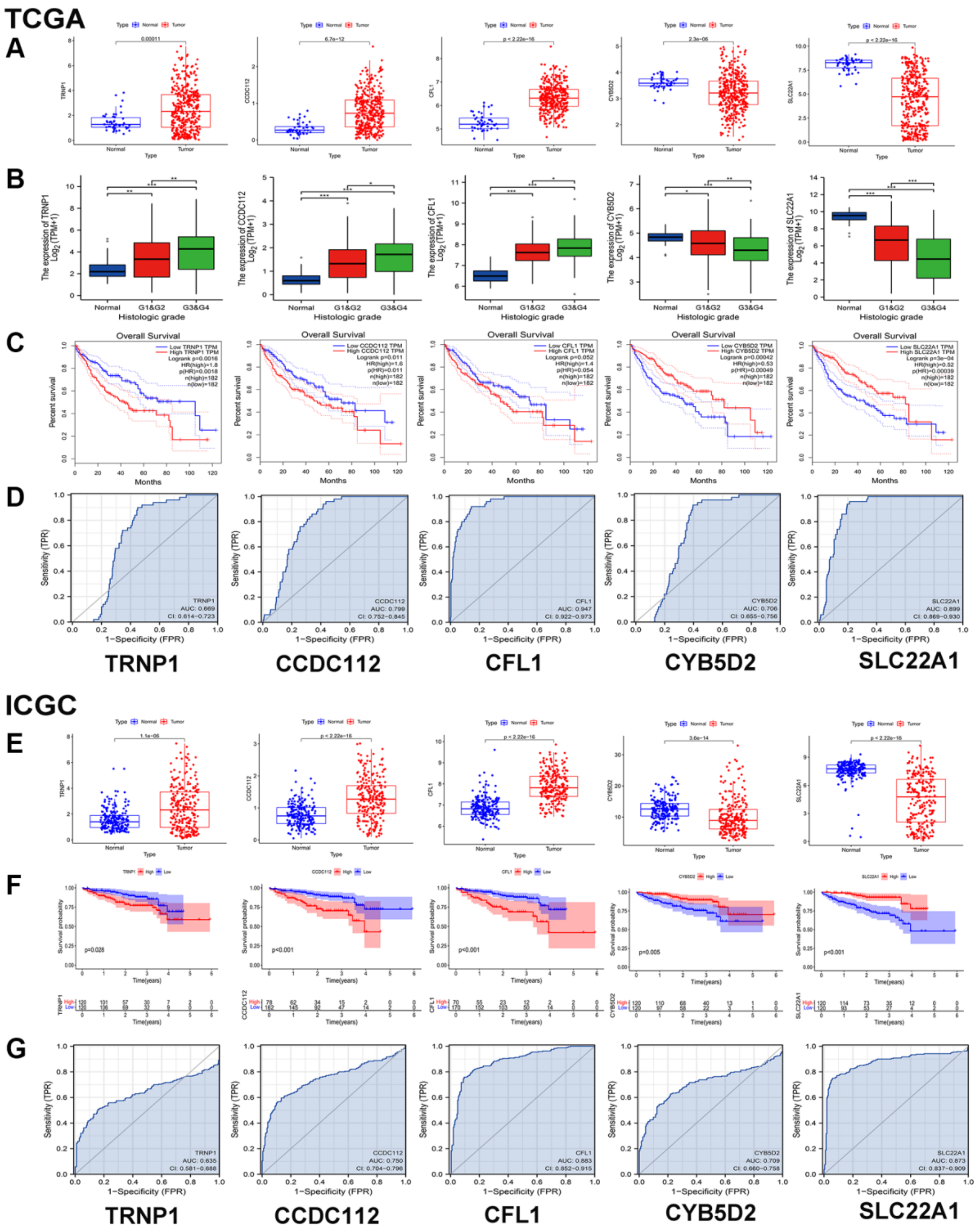

Supplementary Figure 6. The expression of five model genes between tumor and normal tissues in TCGA database (**A**). The expression of five model genes among different pathological grade (**B**) in TCGA database. The K-M curves of five genes in TCGA database (**C**). ROC curves of five genes in TCGA database (**D**). The expression of five model genes between tumor and normal tissues (**E**), the K-M curves five model genes (**F**), the ROC curves of five genes (**G**) in ICGC database.

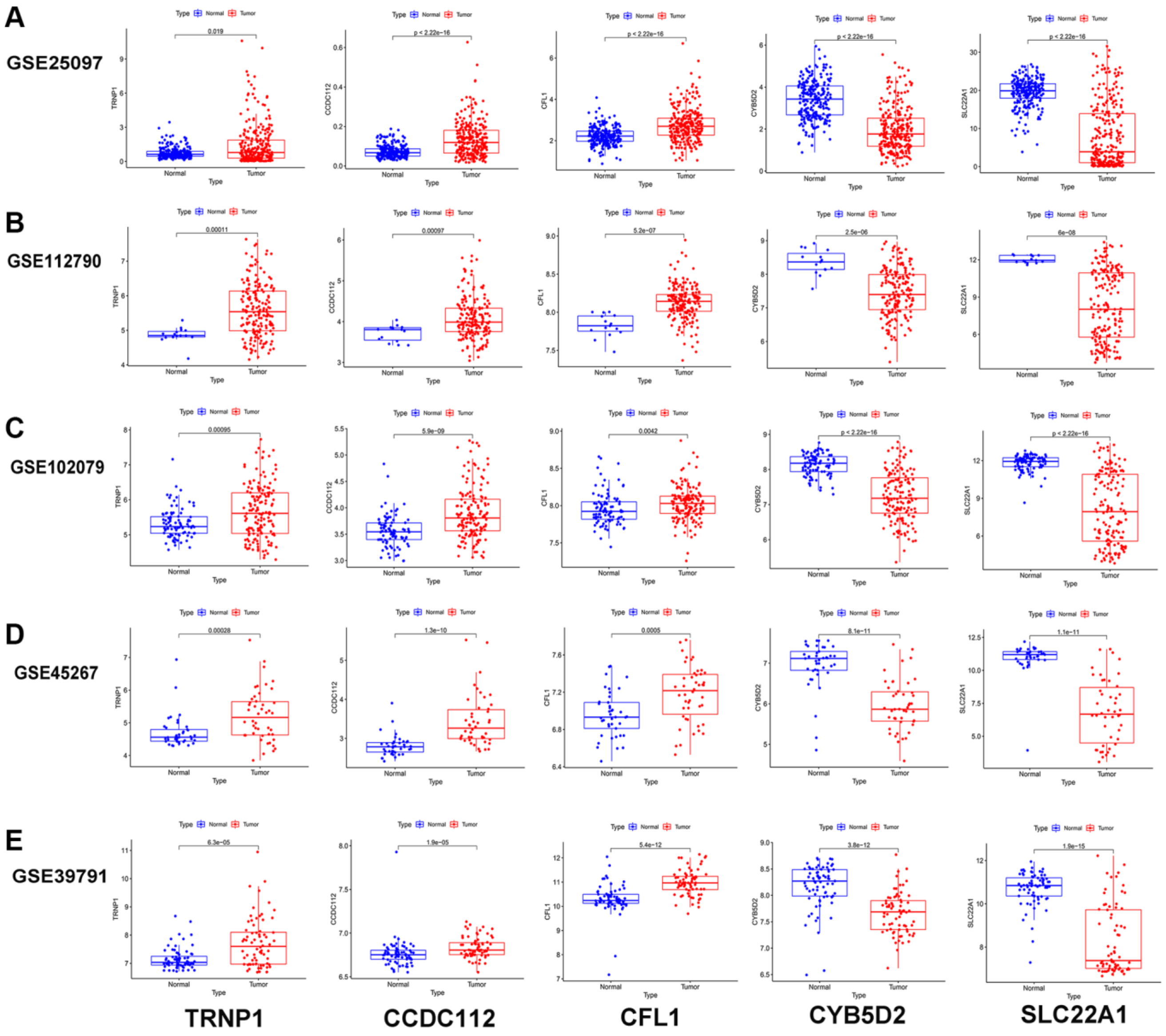

Supplementary Figure 7. Five genes expression in GSE25097 (**A**), GSE112790 (**B**), GSE102079 (**C**), GSE45267 (**D**), GSE39791 (**E**) dataset.

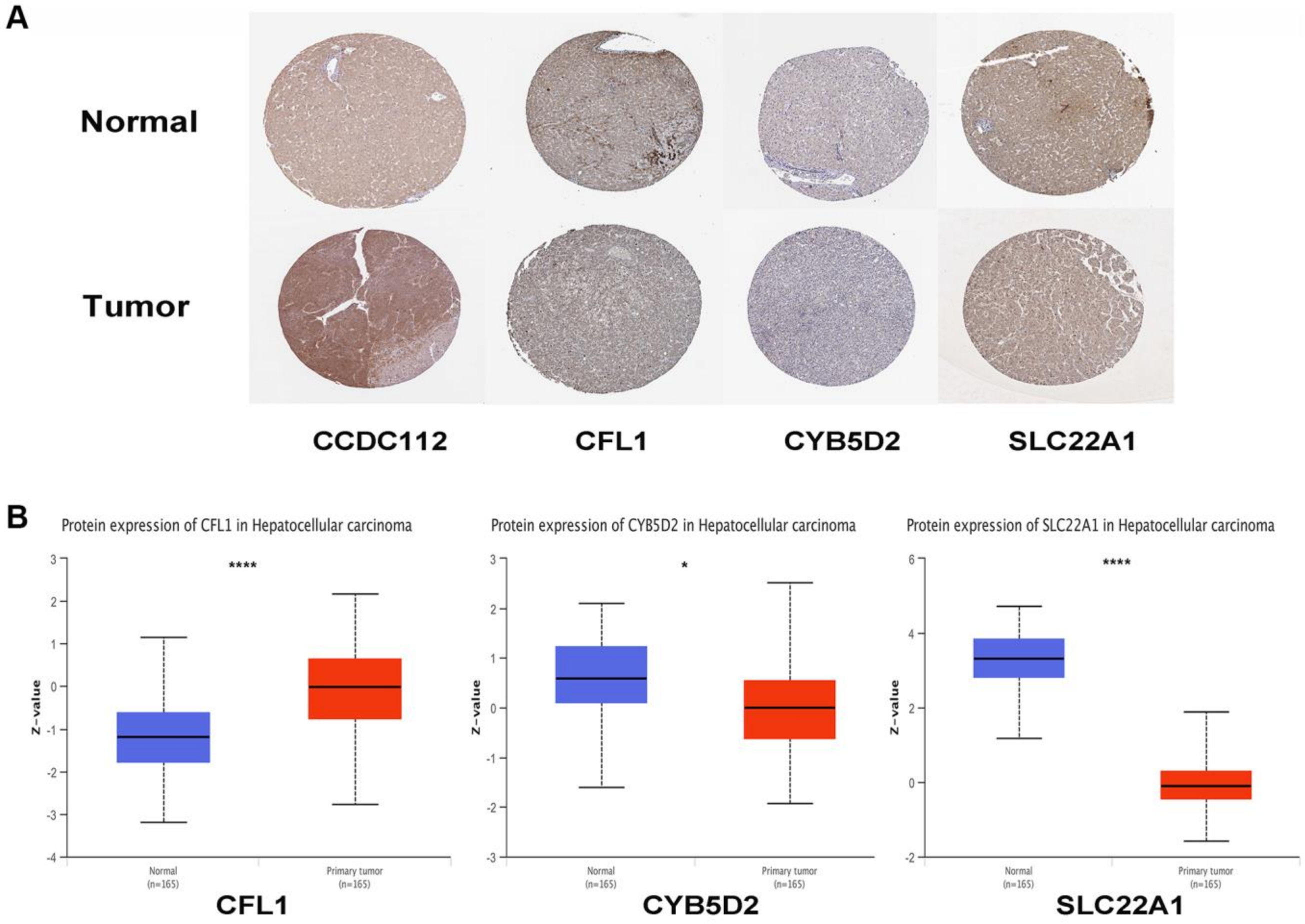

Supplementary Figure 8. Immunohistochemistry analysis of CCDC112, CFL1, CYB5D2, SLC22A1 expression in HPA database (**A**); Protein levels of CFL1, CYB5D2, SLC22A1 in CPTAC database (**B**).

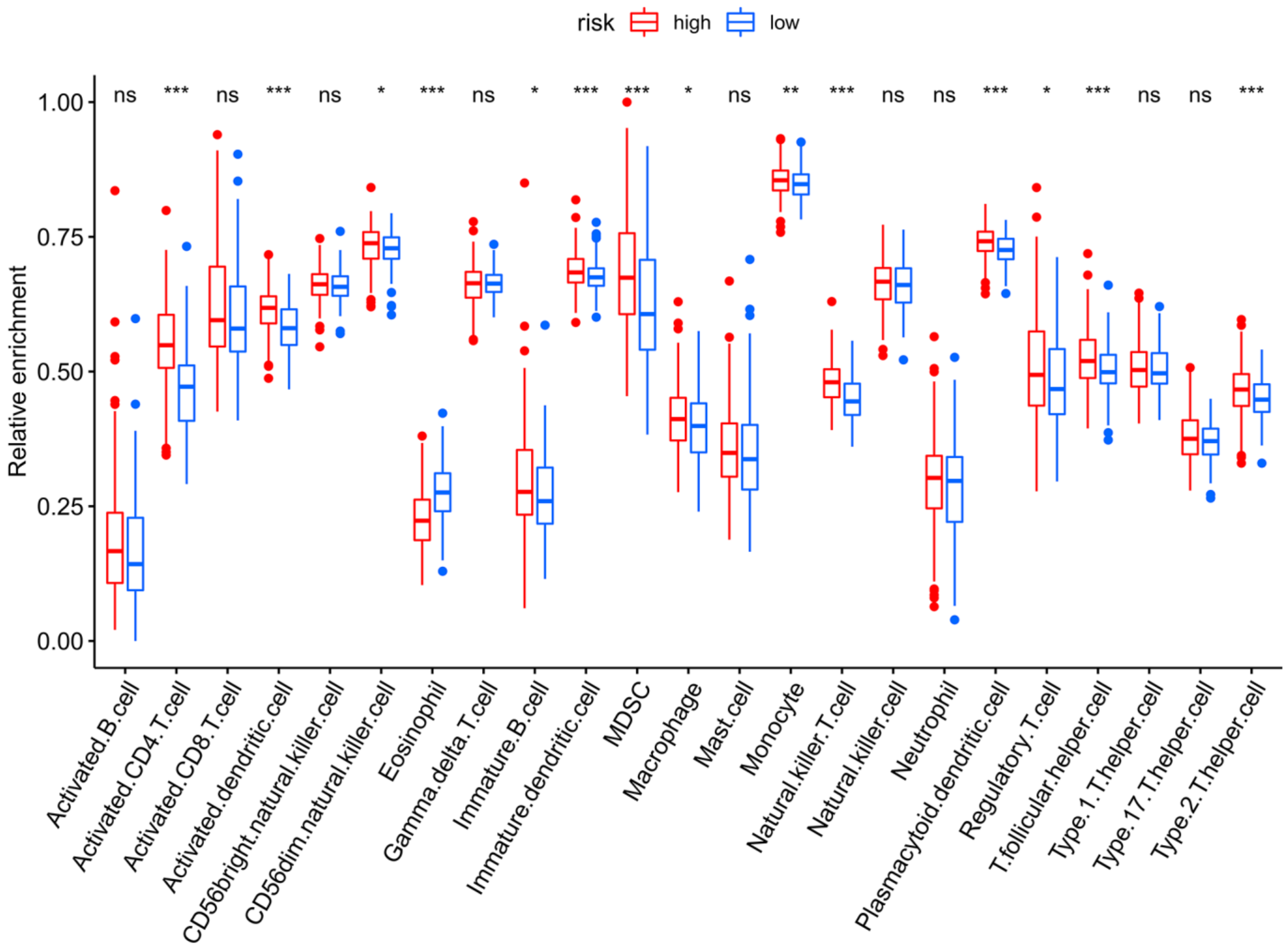

Supplementary Figure 9. The infiltration levels of 23 kinds of immune cells of TME in the two risk groups.
