# Supplementary material for: Identification of fatty acid metabolism-related clusters and immune infiltration features in hepatocellular carcinoma
Source: Aging (Albany NY). 2023 Mar 6;15(5):1496–523. doi: 10.18632/aging.204557 (PMC10042688; doi:10.18632/aging.204557)
Supplement: Supplementary Tables [file aging-15-204557-s002.pdf]

SUPPLEMENTARY TABLES

Supplementary Table 1. Fatty acid metabolism-related genes.

Gene symbol	Description	Category	Relevance score
FASN	Fatty Acid Synthase	Protein Coding	88.04946899
FABP2	Table-S1:Fatty Acid Binding Protein 2	Protein Coding	80.34545898
LIPA	Lipase A, Lysosomal Acid Type	Protein Coding	79.07958984
INS	Insulin	Protein Coding	78.31687164
SLC17A5	Solute Carrier Family 17 Member 5	Protein Coding	75.35491943
FADS1	Fatty Acid Desaturase 1	Protein Coding	73.69827271
HADHA	Hydroxyacyl-CoA Dehydrogenase Trifunctional Multienzyme Complex Subunit Alpha	Protein Coding	73.44226837
FABP4	Fatty Acid Binding Protein 4	Protein Coding	72.57804871
PPARG	Peroxisome Proliferator Activated Receptor Gamma	Protein Coding	70.81949615
FABP1	Fatty Acid Binding Protein 1	Protein Coding	70.25164032
CPT2	Carnitine Palmitoyltransferase 2	Protein Coding	69.19077301
ACADM	Acyl-CoA Dehydrogenase Medium Chain	Protein Coding	66.55280304
MTR	5-Methyltetrahydrofolate-Homocysteine Methyltransferase	Protein Coding	65.85922241
ALB	Albumin	Protein Coding	65.84506226
FADS2	Fatty Acid Desaturase 2	Protein Coding	65.0801239
FABP3	Fatty Acid Binding Protein 3	Protein Coding	63.80797577
DDC	Dopa Decarboxylase	Protein Coding	62.60398865
FA2H	Fatty Acid 2-Hydroxylase	Protein Coding	62.45428848
PPARA	Peroxisome Proliferator Activated Receptor Alpha	Protein Coding	61.77651215
BAAT	Bile Acid-CoA:Amino Acid N-Acyltransferase	Protein Coding	61.54239273
CPT1A	Carnitine Palmitoyltransferase 1A	Protein Coding	61.52598572
ACADVL	Acyl-CoA Dehydrogenase Very Long Chain	Protein Coding	61.33808136
HADHB	Hydroxyacyl-CoA Dehydrogenase Trifunctional Multienzyme Complex Subunit Beta	Protein Coding	60.69838715
MMACHC	Metabolism Of Cobalamin Associated C	Protein Coding	60.51202393
ABCD1	ATP Binding Cassette Subfamily D Member 1	Protein Coding	60.29699326
LPL	Lipoprotein Lipase	Protein Coding	58.79434204
FABP5	Fatty Acid Binding Protein 5	Protein Coding	57.8329277
FAAH	Fatty Acid Amide Hydrolase	Protein Coding	57.01290512
PNPLA3	Patatin Like Phospholipase Domain Containing 3	Protein Coding	56.81323242
CYP2D6	Cytochrome P450 Family 2 Subfamily D Member 6	Protein Coding	55.99719238
AKR1D1	Aldo-Keto Reductase Family 1 Member D1	Protein Coding	54.9125061
CYP2C19	Cytochrome P450 Family 2 Subfamily C Member 19	Protein Coding	53.54149628
NR1H4	Nuclear Receptor Subfamily 1 Group H Member 4	Protein Coding	53.41639328
ACADS	Acyl-CoA Dehydrogenase Short Chain	Protein Coding	53.31412506
ACOX1	Acyl-CoA Oxidase 1	Protein Coding	52.61158371
ADIPOQ	Adiponectin, C1Q And Collagen Domain Containing	Protein Coding	52.42185211
SCD	Stearoyl-CoA Desaturase	Protein Coding	52.2116394
SLC27A1	Solute Carrier Family 27 Member 1	Protein Coding	52.18554688
FABP12	Fatty Acid Binding Protein 12	Protein Coding	52.18073273
FABP6	Fatty Acid Binding Protein 6	Protein Coding	52.11978912
CD36	CD36 Molecule	Protein Coding	52.04457474
LIPC	Lipase C, Hepatic Type	Protein Coding	51.4848938
APOB	Apolipoprotein B	Protein Coding	50.64818192

APOE	Apolipoprotein E	Protein Coding	50.5888176
PHYH	Phytanoyl-CoA 2-Hydroxylase	Protein Coding	50.57295227
HADH	Hydroxyacyl-CoA Dehydrogenase	Protein Coding	50.49472809
CYP3A4	Cytochrome P450 Family 3 Subfamily A Member 4	Protein Coding	50.4631424
AMACR	Alpha-Methylacyl-CoA Racemase	Protein Coding	50.12758636
HSD17B4	Hydroxysteroid 17-Beta Dehydrogenase 4	Protein Coding	50.03477859

Supplementary Table 2. 79 prognostic genes identified by unicox analysis.

ID	HR	HR.95L	HR.95H	*p* value
CYP3A4	0.778076355	0.631093988	0.95929105	0.018822658
RANBP3L	0.485929735	0.257884755	0.915632671	0.025573005
SORD	0.553252858	0.315242258	0.970963495	0.039146754
SEC14L2	0.556356443	0.35219986	0.878854671	0.011952572
AQP9	0.677623041	0.493691285	0.930081205	0.016015165
ALAS1	0.341885541	0.131021095	0.892113769	0.028289079
ADH1B	0.605729859	0.438281266	0.837153423	0.002392409
ECM2	0.490288961	0.28657424	0.838816724	0.009282406
PACRG	0.489240618	0.272397527	0.87870248	0.016722256
GRHPR	0.320862856	0.105618949	0.97475854	0.044956842
APOC4	0.564618825	0.325778952	0.978560512	0.041629443
ZNF385B	0.599719641	0.383262564	0.938426242	0.025210962
MYRIP	0.654531516	0.44584435	0.960899259	0.030497356
UNC119	2.406159309	1.076484474	5.378249999	0.032390758
COBLL1	0.401391037	0.187490206	0.859323633	0.018756828
CYP3A43	0.494188942	0.307679981	0.79375561	0.003553024
ST6GALNAC4	4.577439148	2.318914107	9.035672813	1.16456E-05
TMSB10	3.52740543	1.218053353	10.21514291	0.020149295
TRNP1	2.23135374	1.514507558	3.287497302	4.9203E-05
KCTD17	2.14699596	1.313742559	3.50874806	0.002297419
ADI1	0.209793466	0.067094199	0.655992608	0.007257318
CD24	1.433969982	1.041136815	1.975023724	0.027329796
GTF3C6	13.57443192	3.603410607	51.13633223	0.000116072
PAFAH1B3	2.512457785	1.407123335	4.486063136	0.001841278
PDK4	0.541413324	0.326464404	0.897887746	0.017440487
IKBKE	2.254088928	1.312611493	3.87084596	0.003219696
ANKRD13D	3.523342088	1.528676787	8.120709085	0.003115166
LRRC1	1.808283501	1.125661734	2.904859535	0.014308862
ARPC3	10.79177831	1.894809774	61.4639426	0.007361876
GLTP	17.37701093	3.613247439	83.57039306	0.000366508
SLC2A6	1.715164093	1.051338438	2.798135939	0.030738336
BTNL9	0.366002192	0.201388798	0.665169096	0.000975159
OAZ1	18.94482761	2.560843715	140.1516583	0.003965072
CIB2	1.766576726	1.09180951	2.858367965	0.020464379
SH3BGRL3	6.03881903	1.808128189	20.16855635	0.003471103

NFKBIE	2.896363352	1.295007694	6.477892534	0.009613449
GNAZ	1.915092976	1.255313579	2.92164537	0.002568985
GRAMD1A	2.411861311	1.140248758	5.101584143	0.021259918
PLP2	2.429269296	1.239697358	4.760314505	0.009710398
P2RX4	3.309419925	1.426343897	7.678555126	0.00532122
BAK1	3.838358962	1.703445451	8.648941187	0.001174366
DYNLT1	13.36161281	3.008589083	59.34100404	0.000654496
CAPG	2.309200988	1.383797324	3.853461134	0.001358784
CCDC149	3.732128772	1.652195378	8.430470968	0.001536791
TRAPPC4	14.09480342	4.075780094	48.74244409	2.92156E-05
C11orf80	2.560164894	1.288077049	5.088549859	0.007312492
SMPD2	5.253812801	1.795266114	15.37518518	0.002461619
RGS19	3.177490631	1.340891369	7.529652994	0.008630722
ARPC1B	4.21079628	1.520584487	11.66051966	0.005667732
S100A6	1.83503394	1.073343413	3.13725274	0.026511316
C12orf49	2.691628705	1.383349689	5.237189949	0.003551498
PHLDA2	2.157304562	1.382549384	3.366218252	0.000706858
EGLN3	2.568763817	1.705472772	3.869043035	6.34526E-06
MMP9	1.493898792	1.071488971	2.082833945	0.017924239
RNFT2	3.528666911	1.790133293	6.955621806	0.000270858
NRM	2.327320495	1.207843706	4.484372159	0.011594105
GABARAPL1	0.387683107	0.17874024	0.840874958	0.016453825
GLS	2.339010916	1.280372041	4.272954962	0.005712296
PDE4A	1.807937851	1.009799228	3.236919955	0.046285649
TMEM189	6.608824384	2.229300737	19.59204473	0.000659563
ANKRD24	0.30219409	0.136574227	0.668656669	0.003144648
TRPM2	2.771002254	1.427282838	5.37977007	0.002603816
ABHD12	13.09979991	2.834703327	60.5371137	0.000987256
CCDC112	3.57319329	1.807429626	7.064015164	0.000250133
ASRGL1	2.309893898	1.433019387	3.723334011	0.000588197
RBM38	3.92752773	1.482062082	10.408116	0.005937778
CFL1	128.1863798	14.51617911	1131.960955	1.25869E-05
DOK1	2.09453848	1.020148304	4.300444777	0.043976754
IFI27L2	1.761646639	1.025608679	3.025909339	0.040210618
TMED9	8.276218821	1.054130849	64.97845883	0.044420152
SAAL1	6.133981755	1.871604613	20.10346197	0.002745506
MMD	3.088608202	1.519324905	6.278775918	0.001836454
CYB5D2	0.179105725	0.05980064	0.536430053	0.002120822
RRM2	3.054808696	1.703875719	5.476840867	0.000177522
AKR1D1	0.6053703	0.414352169	0.884448612	0.009465756
C16orf45	0.50778156	0.289491837	0.890671447	0.018088686
SLC22A1	0.623023973	0.474713929	0.817669016	0.000646909
FETUB	0.702168912	0.515209768	0.956971726	0.025195346
CD14	0.373053627	0.139944271	0.994460202	0.048716011

Supplementary Table 3. The regression coefficient of five candidate genes.

ID	coef	HR	HR.95L	HR.95H	*p* value
TRNP1	0.382912477	1.466549668	0.954978512	2.25216369	0.080206219
CCDC112	0.650210449	1.915943995	0.921571858	3.98323946	0.081641119
CFL1	1.885657032	6.59068331	0.509098487	85.32161766	0.148951447
CYB5D2	−1.230989748	0.292003425	0.09700108	0.879021141	0.028575575
SLC22A1	−0.290320823	0.748023546	0.557853321	1.003022129	0.052403777
